# Surgical Management of a Large Endodontic Periapical Lesion With Bone Putty and Platelet-Rich Fibrin: A Case Report With a Two-Year Volumetric Follow-Up

**DOI:** 10.7759/cureus.69355

**Published:** 2024-09-13

**Authors:** Rajat Sharma, Monika Tandan, Sonal Soi, Alpa Gupta

**Affiliations:** 1 Conservative Dentistry and Endodontics, Manav Rachna Dental College, Faridabad, IND

**Keywords:** bone putty, itk-snap, palatal perforation, periradicular surgery, platelet-rich fibrin, volumetric analysis

## Abstract

The present case report evaluates the surgical management of a large periapical lesion with palatal perforation using platelet-rich fibrin (PRF) and bone putty material, with a two-year follow-up. A 15-year-old male presented with persistent swelling and pain in the right maxillary anterior region, having a history of trauma and recurrent swelling. Cone beam computed tomography (CBCT) imaging revealed a large periapical lesion extending from teeth #11 to #15 with a palatal breach. Initial non-surgical root canal treatment (RCT) failed due to persistent exudation from teeth #11, #12, and #13. This led to the decision for periradicular surgery involving cystic lesion enucleation, apicoectomy, retrofill with mineral trioxide aggregate (MTA), and placement of bone putty and PRF. Follow-up assessments, including volumetric analysis with ITK-SNAP software (www.itksnap.org), showed a 97% reduction in lesion volume, from 4055 to 132 cubic millimeters. The palatal perforation was successfully repaired, with no clinical symptoms reported. Substantial reduction in lesion size and successful repair of the perforation highlight the potential of this approach in complex surgical endodontic cases.

## Introduction

Most periapical radiolucent lesions can typically be resolved with standard root canal treatment (RCT) [1]. However, periradicular surgery is indicated if RCT has failed or when orthograde RCT cannot be completed due to persistent exudation into the root canal despite repeated chemo-mechanical debridement [2]. The success of endodontic therapy ranges from 53% to 98% when performed for the first time [3], while the success rate for retreatment cases with periapical lesions is lower [4].

Bone defects after apicoectomy are often large and complicated, resulting from extensive apical lesions, endo-periodontal lesions, and through-and-through lesions [5]. In cases involving large through-and-through lesions associated with labial and palatal perforations of endodontic origin, the effectiveness of the surgical intervention depends on several factors, including appropriate case selection, the surgical proficiency of the practitioner, and the materials used to seal the enucleated periapical defect [6]. In such lesions, a 25% healing rate has been documented [7]. Based on limited evidence, a systematic review and meta-analysis demonstrated that if a large or through-and-through lesion exists, guided tissue regeneration procedures may lead to better outcomes, with resorbable membranes being more effective than non-resorbable ones [8].

Autologous platelet-rich concentrates, such as platelet-rich plasma and platelet-rich fibrin (PRF), are effective agents for bone regeneration [9]. However, if these concentrates are not properly supported by scaffolds, they may push through palatal perforations in large periapical lesions, making it challenging to maintain their precise placement. Therefore, in addition to platelet-rich concentrates, bone graft/putty materials contribute to the successful healing of such bony defects.

The present case report describes the surgical management of a large periapical lesion of endodontic origin associated with a palatal perforation using platelet-rich fibrin and bone putty material with a two-year follow-up. Healing was evaluated by ITK-SNAP software (www.itksnap.org) for cone beam computed tomography (CBCT) analysis.

## Case presentation

**Figure 1:**
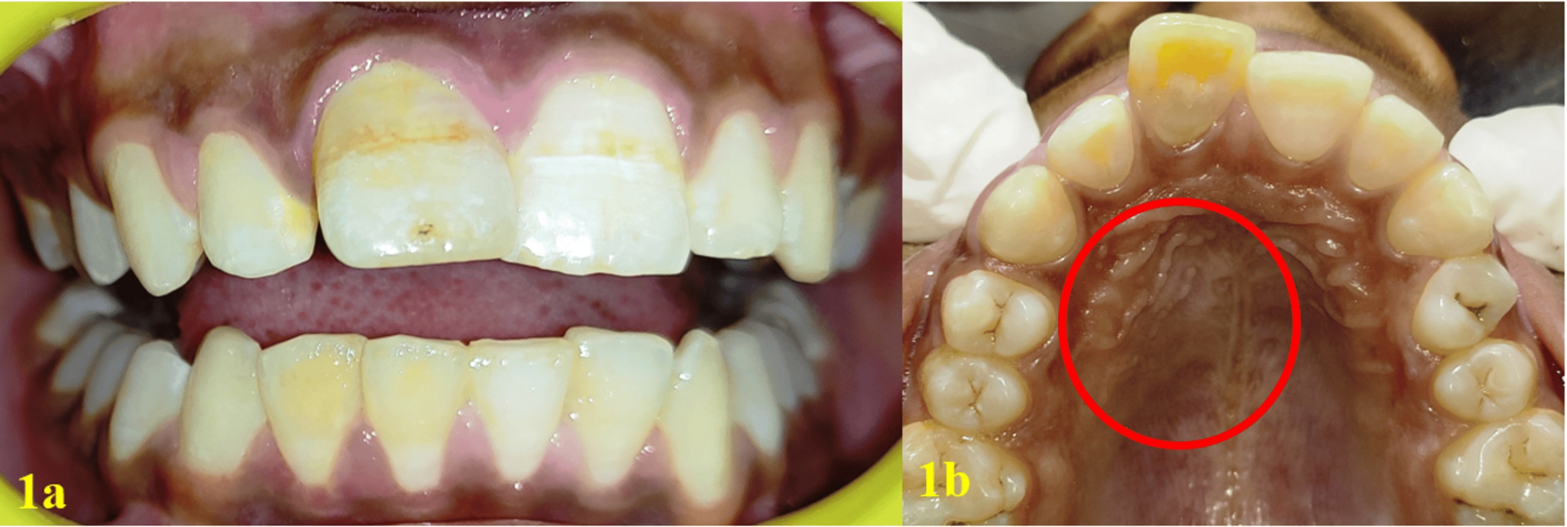
Labial and palatal view on intra-oral examination 1a: Discolored and labially inclined right central incisor are noted; 1b: Palatal swelling was observed in the region of teeth #11, #12, and #13.

**Figure 2:**
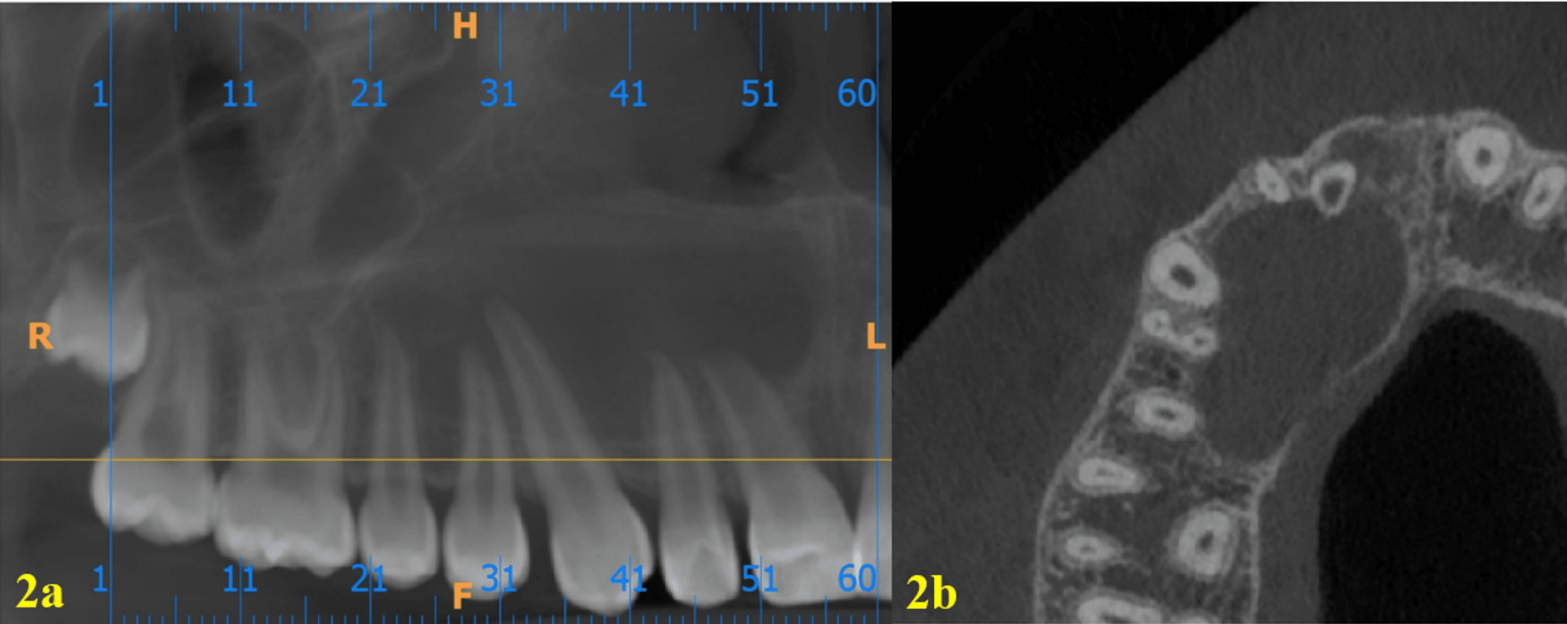
Cone-beam computed tomography (CBCT) findings 2a. A large, well-defined periapical lesion with a well-defined bony outline affecting teeth #11, #12, #13, #14, and #15; 2b. A breach in the continuity of the palatal cortical plate is noted.

**Figure 3:**
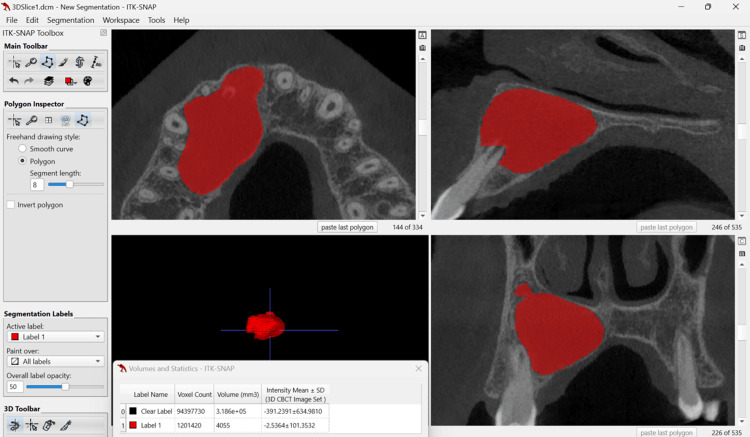
Volumetric analysis conducted using ITK-SNAP software shows a periapical lesion volume of 4055 cubic millimeters ITK-SNAP software: www.itksnap.org

A 15-year-old male patient reported to the department of conservative dentistry and endodontics in March 2022, with the chief complaint of pain in the right upper lip region and swelling on the right cheek and palatal region. The swelling had persisted for five months, and it continued to increase. He had taken over-the-counter (OTC) medications multiple times, but the swelling persisted. The pain became severe in the last three days and was not relieved by the medications prescribed by a dental health professional. The patient had a history of trauma to the upper front teeth five to six years ago, which was followed by swelling in the upper lip for 15 days. The swelling subsided after taking medications from a nearby primary health center. There was no history of fever or weight loss, and no significant medical history was obtained. An extra-oral clinical examination revealed diffuse hard swelling in the right maxillary anterior region, lateral to the nose. Intra-oral examination showed a discolored and labially inclined right central incisor, as well as a swelling on the palate in the region of teeth #11, #12, and #13 (Figures 1a, 1b). The swelling was oval-shaped and diffuse, extending from the distal aspect of tooth #11 to the mesial aspect of tooth #14. It was hard, non-tender, and non-fluctuant. Electric pulp testing (EPT) indicated that teeth #11, #12, #13, #14, and #15 were non-responsive. Based on the history and clinical findings, a provisional diagnosis of pulp necrosis with symptomatic apical periodontitis (according to diagnostic terminology approved by the American Association of Endodontists and the American Board of Endodontics) involving teeth #11, #12, #13, #14, and #15 was made. The patient presented with CBCT reports, which had been advised by a dental health professional three days prior. The CBCT revealed a large, well-defined periapical lesion with a well-defined bony outline in relation to teeth #11, #12, #13, #14, and #15, with a breach in the continuity of the palatal cortical plate in the right maxillary anterior region (Figures 2a, 2b). Volumetric analysis using ITK-SNAP software showed a lesion volume of 4055 cubic millimeters (Figure 3).

Endodontic procedure

**Figure 4:**
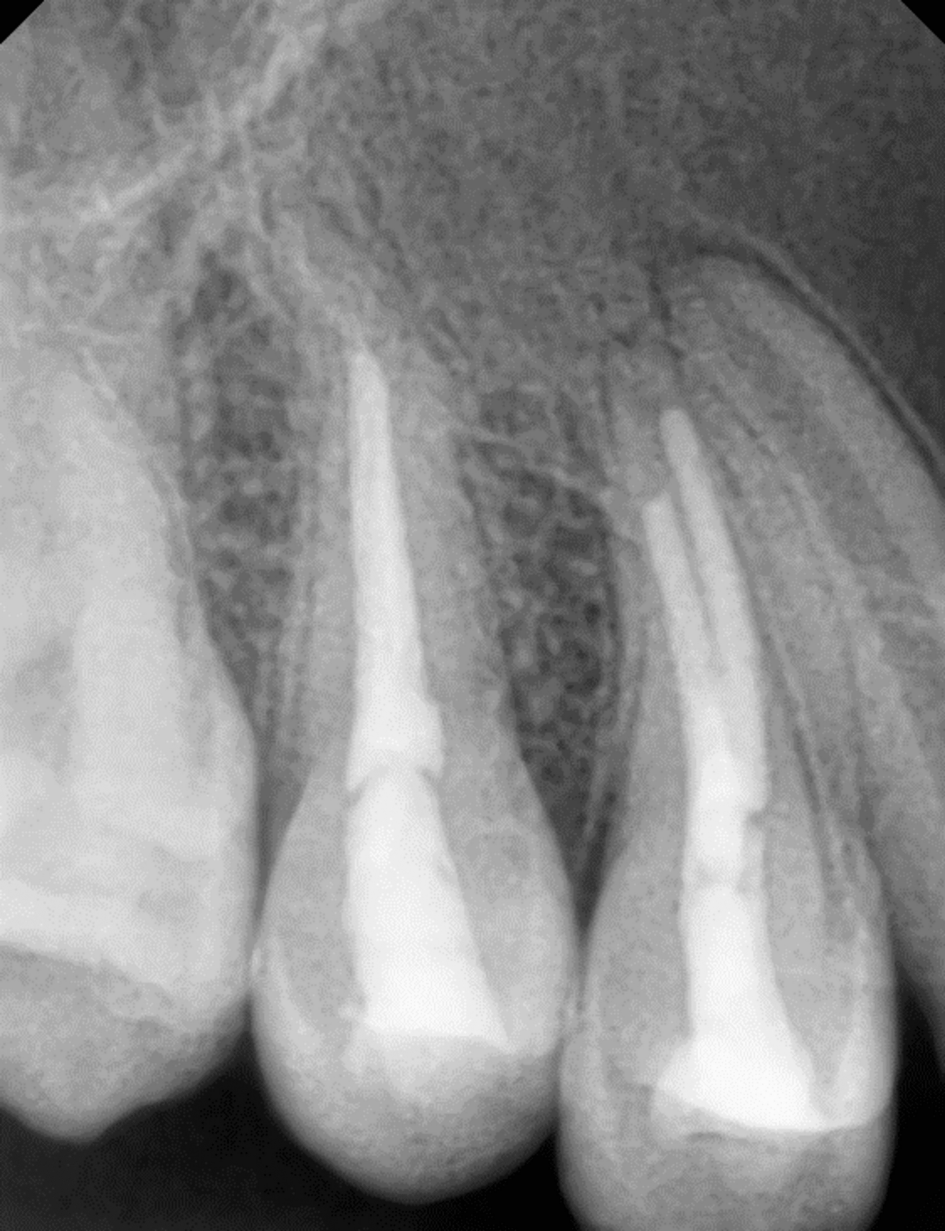
Root canal treatment Obturation performed on teeth #14 and #15

A treatment plan was initially developed for non-surgical RCTs on teeth #11, #12, #13, #14, and #15. The procedural steps were explained to the patient, and written informed consent was obtained. After applying a rubber dam, access openings were made using a large round (314 G) bur (SS White Burs, Inc., Lakewood, NJ), and working length was estimated using a size 10 K file. Chemo-mechanical preparation was performed with hand K files and Ni-Ti rotary files (Dentsply Maillefer, Ballaigues, Switzerland). Canals were irrigated with 5.25% sodium hypochlorite (CanalPro, Coltene, Switzerland), normal saline (0.9% sodium chloride, Otsuka, India), and 2% chlorhexidine (CanalPro). After drying the canal, a calcium hydroxide intracanal dressing was placed for one week. Root canal obturation of teeth #14 and #15 was completed using Gutta-percha (Dentsply Maillefer) and Bioceramic Sealer (BioRoot RCS, Septodont, Saint-Maur-des-Fosses, France) with lateral condensation technique during the next appointment (Figure 4). However, dry canals could not be obtained in teeth #11, #12, and #13 despite multiple dressings. Consequently, the treatment plan was revised to include periradicular surgery and obturation of teeth #11, #12, and #13 on the day of surgery.

Surgical procedure

**Figure 5:**
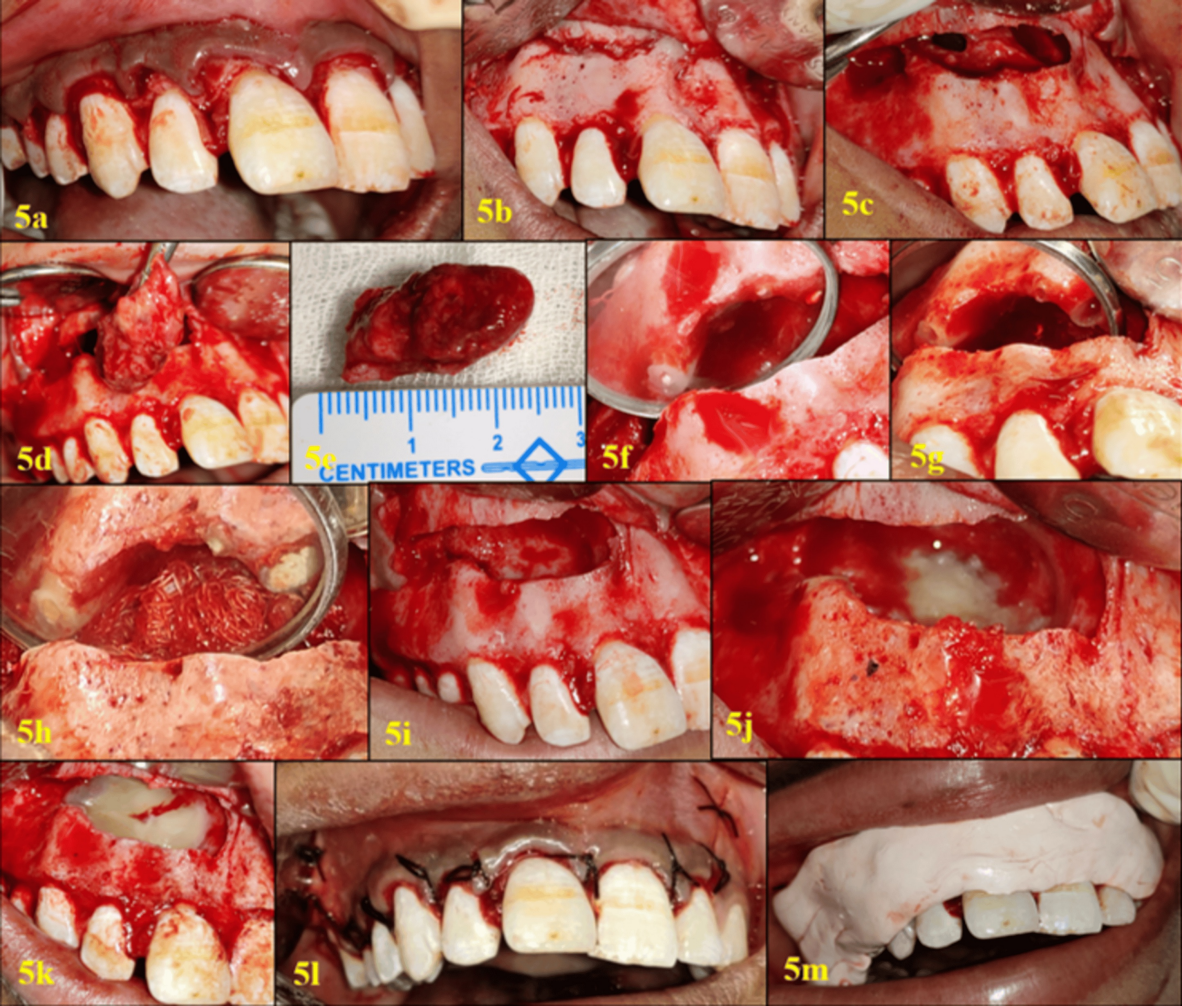
Surgical procedure 5a. Crevicular incision from the distal aspect of #16 to mesial aspect of #22 with bilateral releasing incisions; 5b. Raised mucoperiosteal flap; 5c. A window was created to enucleate the periradicular cyst; 5d. The periradicular cyst was enucleated; 5e. The periradicular cyst was sent for histopathological examination; 5f. Apicoectomy performed on teeth # 11, #12, and #13; 5g. Gutta-percha was removed and retrocavities were prepared in teeth #11, #12, and #13 using retro-ultrasonic tips; 5h. Retrofilling with mineral trioxide aggregate (MTA) completed in teeth #11, #12, and #13 using a microsurgical plugger; 5i. Bony cavity cleaned after enucleation of periradicular cyst and MTA retrofilling; 5j. Palatal perforation was sealed with NovaBone putty material; 5k. Platelet-rich fibrin was placed into the bony cavity till it was filled; 5l. The surgical site closed with multiple interrupted sutures using 4-0 silk; 5m periodontal dressing (Coe-Pak)

**Figure 6:**
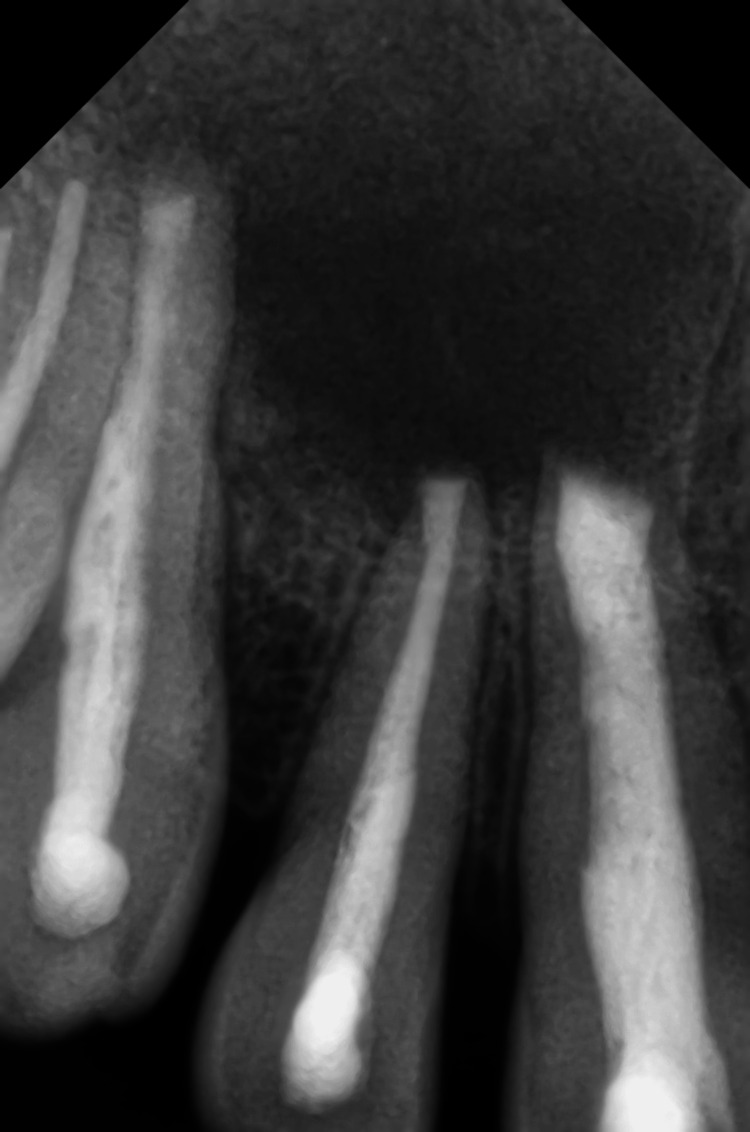
Postoperative intra-oral periapical radiograph (IOPAR) of retrofilled teeth #11, #12, and #13

The patient's surgical region was painted with 5% povidone-iodine and draped under sterile conditions. Local anesthesia was achieved with 2% lignocaine (1:80000 adrenaline) (Lignospan Special, Septodont). A crevicular incision was made from the distal aspect of tooth #16 to the mesial aspect of tooth #22, with releasing incisions given bilaterally (Figure 5a), thus raising a rectangular mucoperiosteal flap (Figure 5b). The cystic lesion was enucleated and sent for histopathological examination (Figures 5c-5e). Bur #701 (SS White Burs, Inc.) was used in a straight handpiece of the physiodispenser (Implantmed, W & H, Bürmoos, Austria) at a slow speed of 40,000 rpm with an adequate water jet to cut 3 mm of root tips of teeth #11, #12, and #13 (Figure 5f). A straight ultrasonic tip No. F00106/F00079 (Satelec Acteon, Mérignac, France) was used to remove gutta-percha and prepare the retrocavity (Figure 5g). After mixing ProRoot mineral trioxide aggregate (MTA) (Dentsply Maillefer) according to the manufacturer’s instructions, MTA was placed into the retrocavity and condensed using a microsurgical retrofilling DE plugger (Hu-Friedy, Chicago, IL) in two to three increments (Figure 5h). Sharp margins were rounded off, and the cavity was thoroughly lavaged with normal saline (Figure 5i). Clinically, the nasal floor remained intact, and the palatal perforation was sealed using bone putty material (NovaBone, NovaBone Products, Bangalore, India) (Figure 5j). To prepare the PRF, venous blood was drawn from the patient into 10 mL sterile tubes without anticoagulants and centrifuged immediately at 3000 rpm for 10 minutes. The fibrin clot formed in the middle layer was then isolated from the red blood cell layer and applied over the bone putty in the osteotomy site, filling the bony cavity completely (Figure 5k). The mucoperiosteal flap was then repositioned, and the surgical site was closed with multiple interrupted sutures using 4-0 silk (Mersilk, Ethicon, Somerville, NJ) (Figure 5l) and a periodontal dressing (Coe-Pak Automix, GC America Inc., Alsip, IL) (Figure 5m). A postoperative intra-oral periapical radiograph (IOPAR) was taken (Figure 6), and a custom-made acrylic palatal splint was fitted in the patient's mouth to support the palatal perforation site. Postoperative instructions were given, and antibiotics and analgesics were prescribed for five days. The patient was recalled after five days for evaluation, palatal splint adjustment, and suture removal. Histopathological examination revealed a cyst wall lined by stratified squamous epithelium, with dense inflammatory infiltrates, including lymphocytes, plasma cells, and histiocytes, as well as inflamed granulation tissue. The presence of cholesterol clefts was noted. No hyaline bodies, metaplasia, calcification, granuloma, or atypia were identified. These findings are consistent with the diagnosis of a periradicular cyst associated with teeth #11, #12, #13, #14, and #15.

**Figure 7:**
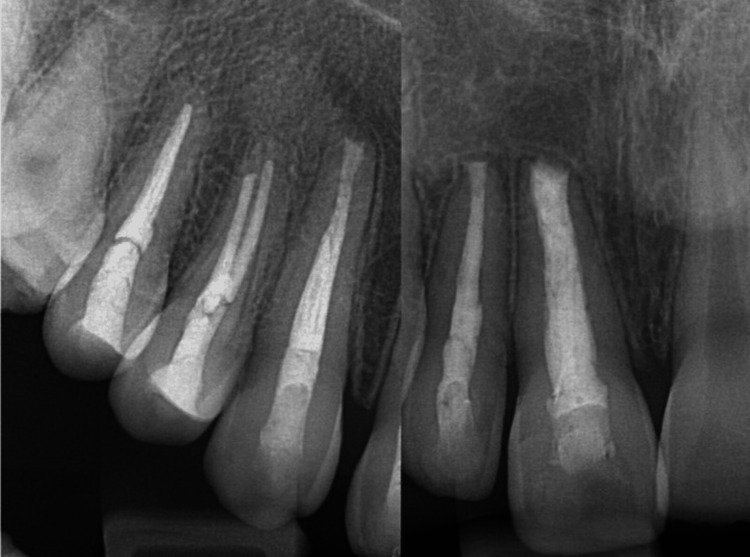
Intra-oral periapical radiograph (IOPAR) performed at the two-year follow-up revealed near-complete healing of the periapical lesion.

**Figure 8:**
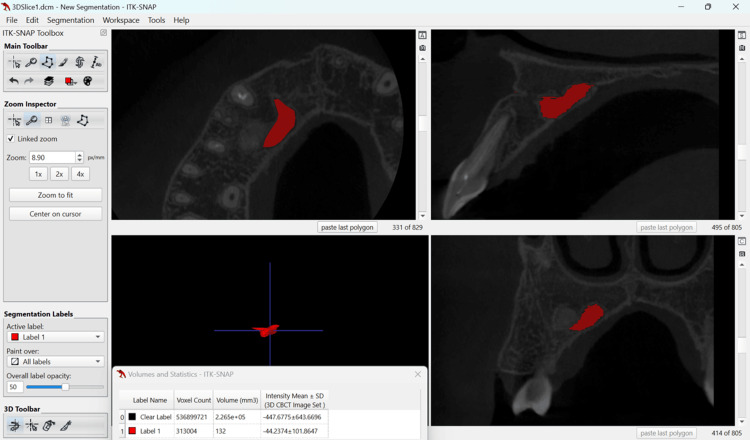
Cone beam computed tomography (CBCT) done at the two-year follow-up showed a reduction in lesion volume to 132 cubic millimeters. ITK-SNAP software: www.itksnap.org

Regular follow-up was maintained, and the periapical healing was assessed using IOPAR and ITK-SNAP software for CBCT volumetric analysis (in terms of reduction in the lesion volume). Postoperative follow-up evaluation after two years revealed near-complete healing of the periapical lesion (Figure 7), with an almost 97% reduction in lesion volume from 4055 cubic millimeters to 132 cubic millimeters (Figure 8).

## Discussion

According to the guidelines for periradicular surgery (2020) provided by the Royal College of Surgeons in collaboration with the British Endodontic Society, the indications for periradicular surgery include: (a) When orthograde root canal treatment cannot be completed due to persistent exudation into the root canal despite repeated chemo-mechanical debridement; (b) When previous treatment has been carried out to guideline standards but symptomatic or progressing periradicular disease persists in an optimally root-filled tooth; (c) Symptomatic or progressing periradicular disease associated with a well root-filled tooth where root canal retreatment has failed, or where retreatment may compromise the structural integrity of the tooth, be destructive to a restoration or fixed prosthesis, or involve the removal of a post with a high risk of root fracture; (d) Symptomatic or progressive periradicular disease associated with a tooth in which iatrogenic or developmental anomalies prevent orthograde root canal treatment; (e) when a biopsy of periradicular tissue is required; and (f) when visualization of the periradicular tissues and tooth root is necessary if perforation or root fracture is suspected [1].

In the present case, the large size of the lesion and persistent pus discharge from teeth #11, #12, and #13, even after multiple appointments, led to the decision to proceed with periradicular surgery. Apicoectomy of teeth #14 and #15 was not performed to preserve the adjacent healthy buccal cortical plate.

There are differing views on the use of collagen membranes and bone grafts in surgical endodontics for treating extensive buccal perforations with periapical diseases [10]. Calcium phosphosilicate (CPS) particles with a bimodal size distribution are combined with binders like glycerine and polyethylene glycol to create the NovaBone putty substance. The binders improve handling characteristics and aid in maintaining particle separation, which facilitates quicker revascularization. After application, the binders dissolve and absorb within 24 to 72 hours, forming a three-dimensional porous scaffold that allows blood and tissue fluids to diffuse throughout the matrix [11]. NovaBone putty has both osteoconductive and osteostimulative properties. Following implantation, the absorption of the graft material and the controlled release of silicon, calcium, and phosphorus ions increase the osteocalcin and alkaline phosphatase levels, which promote new bone growth [12]. Both cellular and non-cellular components are bound within the gel matrix by the silica and calcium-rich surface gel; additionally, hydroxyl carbonate/apatite nucleates crystallize and interact with collagen, glycoproteins, mucopolysaccharides, and osteocellular components [13]. A vital component called carbonate apatite is resorbed by osteoclasts in mildly acidic environments and replaced by new bone via remodeling. It has better osteoconductive qualities than hydroxyapatite (HA) and accelerates osteoblast differentiation alongside the rate at which new bone tissue is deposited. Furthermore, microstructural examination demonstrating new bone growth inside the grafting material indicates that carbonate apatite promotes bone deposition without fibrotic tissue formation [14].

When combined with bone grafts, platelet-rich concentrates help promote improved bone repair. Growth factors such as insulin-like growth factor-1 (IGF-1), platelet-derived growth factor (PDGF), transforming growth factor beta (TGF-β), epidermal growth factor (EGF), vascular endothelial growth factor (VEGF), and basic fibroblast growth factor (FGF) are abundant in PRF and effectively contribute to new bone production [15]. Platelet-rich fibrin functions as a biological link between the bone graft materials and the cytokine release from PRF is crucial for the graft material's ability to self-regulate the inflammatory process [16]. Additionally, PRF demonstrates significant chemotactic and mitogenic potential, promoting cell proliferation, differentiation, angiogenesis, and tissue regeneration, further enhancing bone healing and regeneration [17].

As a newer alternative to PRF, concentrated growth factor (CGF) could also be used, which is a second-generation platelet concentrate prepared by centrifuging blood samples at alternating and controlled speeds using a specialized centrifuge. This method results in a denser fibrin matrix that is richer in growth factors compared to PRF and PRP. The three-dimensional network of fibrin in CGF allows for a slower and more sustained release of growth factors [18].

Given the osteostimulative and osteoconductive qualities of the NovaBone putty material and the characteristics of autologous PRF, it was hypothesized that these factors would work synergistically to manage the extensive periapical lesions with palatal perforations in this case.

For teeth with endodontic involvement, Huumonen and Ørstavik examined alterations in the 2D periapical index (PAI) score to evaluate treatment results and periapical healing [19]. With the increasing use of CBCT in endodontics, lesion volume has been assessed in several studies to gauge periapical healing [20, 21]. Estrela et al. reported that the greatest diameter of each lesion determined the CBCT PAI score [22]. Boubaris et al. introduced a novel volume-based CBCT periapical volume index (PAVI) with scores ranging from 1 to 6 in 2021 [23]. In the current case, the lesion is rated at 6 (>100 mm³) according to the newly developed CBCT PAVI, indicating that a thorough volumetric study is required to obtain meaningful results. The effective lesion volume decrease was computed using ITK-SNAP software version 4.0.1, an open-source image analysis technique supported by the US National Library of Medicine [24]. The bubble cluster and polygonal form fill methods were considered, with the latter being chosen for its higher accuracy and reproducibility.

## Conclusions

This case report demonstrates that the use of PRF and bone putty material effectively managed a large periapical lesion with a palatal perforation. The treatment resulted in a 97% reduction in lesion volume over two years and successful repair of the palatal defect. This outcome underscores the efficacy of integrating advanced biomaterials in complex endodontic surgical cases and highlights the importance of precise case selection and skilled surgical intervention in achieving successful outcomes. Future studies should continue to explore and validate these techniques to further improve the management of challenging periapical lesions.
